# ‘1-8 interferon inducible gene family’: putative colon carcinoma-associated antigens

**DOI:** 10.1038/sj.bjc.6604061

**Published:** 2007-12-11

**Authors:** B Tirosh, V Daniel-Carmi, L Carmon, A Paz, G Lugassy, E Vadai, A Machlenkin, E Bar-Haim, M-S Do, I S Ahn, M Fridkin, E Tzehoval, L Eisenbach

**Affiliations:** 1 Department of Immunology, The Weizmann Institute of Science, Rehovot, Israel; 2 Department of Organic Chemistry, The Weizmann Institute of Science, Rehovot, Israel; 3 Department of Urology, Barzilai Medical Center, Ashkelon, Israel; 4 Department of Hematology, Barzilai Medical Center, Ashkelon, Israel; 5 School of Life and Food Science, Handong University, Pohang, Korea

**Keywords:** cytotoxic T lymphocyte, colon cancer, peptides, tumour immunity

## Abstract

D^b−/−^x*β*2 microglobulin (*β*2m) null mice transgenic for a chimeric HLA-A2.1/D^b^-*β*2m single chain (HHD mice) are an effective biological tool to evaluate the antitumour cytotoxic T-lymphocyte response of known major histocompatibility-restricted peptide tumour-associated antigens, and to screen for putative unknown novel peptides. We utilised HHD lymphocytes to identify immunodominant epitopes of colon carcinoma overexpressed genes. We screened with HHD-derived lymphocytes over 500 HLA-A2.1-restricted peptides derived from colon carcinoma overexpressed genes. This procedure culminated in the identification of seven immunogenic peptides, three of these were derived from the ‘human *1-8D* gene from interferon inducible gene’ (*1-8D*). The *1-8D* gene was shown to be overexpressed in fresh tumour samples. The three *1-8D* peptides were both antigenic and immunogenic in the HHD mice. The peptides induce cytotoxic T lymphocytes that were able to kill a colon carcinoma cell line HCT/HHD, *in vitro* and retard its growth *in vivo*. One of the peptides shared by all the *1-8* gene family primed efficiently normal human cytotoxic T lymphocyte precursors. These results highlight the *1-8D* gene and its homologues as putative immunodominant tumour-associated antigens of colon carcinoma.

Supplementary approaches to surgery and chemotherapy promise better treatment to metastases. One such approach is specific active immunotherapy aimed at induction of anti-tumour cytotoxic T lymphocytes (CTL) (Eisenbach *et al*, 2000). Upon appropriate vaccination, CTL constitute powerful effectors against tumours that present on their cell-surface major histocompatibility (MHC) class I molecules with bound tumour-associated antigenic (TAA) peptides (Eisenbach *et al*, 2000).

Different types of TAA have been identified. Antigens that are uniquely expressed in tumours (Luftl *et al*, 2004); antigens derived from differentiation markers (Panelli *et al*, 2000); antigens arise from mutations that are common in tumours (McArdle *et al*, 2000); antigens from a viral source (Youde *et al*, 2004); and self-antigens that are overexpressed in tumours (Bar-Haim *et al*, 2004). In the latter category, although strong immune reactions against this type of antigens might result in the destruction of normal tissues, experience with peptide immunisation has not demonstrated high toxicity in humans and animals (Gross *et al*, 2004).

Thus far, little is known about target antigens for CTL in colorectal carcinoma. The few potential targets belong to the family of self overexpressed genes, such as *her-2/neu*, *CEA* and *Ep-CAM* (Melief *et al*, 2000). Therefore, there is a strong need to expand the armoury against colon carcinoma by discovering new TAAs (Nagorsen *et al*, 2000). Since the repertoire of peptides eluted from surface MHC class I molecules is highly similar between normal colon and colon tumours (Melief *et al*, 2000), we focused our efforts to identify novel colon carcinoma TAAs from colon carcinoma overexpressed genes.

We utilised the data of Zhang *et al* (1997) comparing transcripts of colon tumour tissue samples and normal tissues excised from the same patients. This study yielded a set of 26 overexpressed genes that are expressed in tumours at least five-fold higher than in normal tissue.

We used the D^b^ × *β*2 microglobulin (*β*2m) null mice, transgenic for a recombinant HLA-A2.1/D^b^-*β*2m single chain (HHD mice), in order to identify only immunologically relevant MHC class I-restricted peptides. These mice mount only HLA-A2.1-restricted CTL responses and were demonstrated as a useful biological tool for identifying potential TAA HLA-A2.1-restricted epitopes (Firat *et al*, 1999; Carmon *et al*, 2000; Machlenkin *et al*, 2005).

We applied the HHD model and showed that from over 500 putative TAA peptides derived from the 26 overexpressed genes in colon carcinoma, seven peptides were antigenic and immunogenic in HHD mice. Three of the seven were derived from ‘human 1-8D from interferon inducible gene’ (*1-8D* gene, *IFITM2*). These peptides elicit a CTL response against a colon carcinoma cell line *in vitro* and *in vivo*. One of the peptides shared by all members of the *1-8* interferon inducible gene family was highly immunogenic in human peripheral blood mononuclear cells (PBMCs). These results highlight *1-8D* gene and its family as a novel colon carcinoma TAA.

## MATERIALS AND METHODS

### Mice

The derivation of HLA-A2.1/D^b^-*β*2 monochain, transgenic, H-2D^b^x*β*2m double-knockout mice (named HHD mice) has been described by Pascolo *et al* (1997). CD1-nude mice were bred in the Weizmann Institute of Science (Rehovot, Israel). All protocols involving animals were in compliance with the Institutional Animal Care and Use Committee and in accordance with the guidelines of the Weizmann Institute of Science institutional animal care and use committee (IACUC). All the experiments, methods and facilities have been accredited by Ministry of Health: The Council for Experimentation on Animals, National Institute of Health: Office of Laboratory Animal Welfare and by Association for Assessment and Accreditation of Laboratory Animal Care (AAALAC). These accreditations meet the standards required by the UKCCCR guidelines.

### Antibodies

BB7.2 and B9.12 are mouse monoclonal antibodies against HLA-A2 (Pascolo *et al*, 1997). CTLA-4-Ig fusion was used to detect surface expression of B7.1.

### Cell lines

HCT-15, HCT/HHD and HCT/HHD/B7.1 were maintained in DMEM containing 10% FCS, combined antibiotics, 2 mM glutamine. RMA-S, RMA-S/HHD/B7.1, T2, EL4/HHD (provided by Dr FA Lemonnier, Pasteur Institute, Paris, France), EL4/HHD/1-8D-Myc were grown in RPMI containing 10% FCS, combined antibiotics, 2 mM glutamine, 5 × 10^−5^ M *β*ME and 50 *μ*g ml^−1^ hygromycin.

### Preparation of tumour extract peptides

Total acid-extracted peptides of colon tumour or of normal adjacent tissues were prepared from a pool of six post-surgical colon cancer specimens. Non-necrotic (1–2 cm) tumour masses were homogenised in PBS, 0.5% Nonidet P-40, 10 *μ*g ml^−1^ soybean trypsin inhibitor, 5 *μ*g ml^−1^ leupeptine, 8 *μ*g ml^−1^ aprotonin and 0.5 mM PMSF and homogenised using a glass-teflon homogeniser. Following further stirring for 30 min at 4°C, the homogenates were titrated with 10% TFA to a final concentration of 0.1% TFA and stirred for another 30 min at 4°C. After ultracentrifugation for 30 min at 42K r.p.m., the supernatants were applied to Sephadex G25 columns and fractions were monitored at OD 230 nm. Peptide fractions below 10 kDa were pooled, lyophilised and further fractionated by Centripep 3 centrifugation (Amicon, Beverly, MA, USA). Lyophilised samples were dissolved in sterile double distilled water, freed from TFA by repeated lyophilisation, and the relative concentration was assessed by measuring the OD at 230 nm. Following lyophilisation, the peptide pool was dissolved in optiMEM (Life Technologies, Paisley, UK) at 30–50 OD at 230 nm per ml for further use.

### Peptide synthesis

Peptides were synthesised on an ABIMED AMS 22 multiple peptide synthesiser (Abimed, Langenfeld, Germany), employing Fmoc strategy. Crude peptides were purified to homogeneity by semipreparative RP-HPLC. For the screening experiments, peptides were synthesised by multi-parallel synthesis (MPS) method adapted to 96-well plates, as was developed by Peptor Ltd (Rehovot, Israel). The peptides were purified by solid-phase extraction over C-18 Sep-Pak resin and analysed by VG platform, API-ESI MS (Fisons, UK). The average purity of the MPS peptides was about 80% as was analysed by RP-HPLC.

### Vaccination

HHD mice were immunised i.p. three times at 7-day intervals with 2 × 10^6^ irradiated (5000 rad) tumour cells, or irradiated peptide-loaded RMA-S/HHD/B7.1 transfectants. Spleens were removed on day 10 after the last immunisation, and splenocytes were restimulated *in vitro*, either with irradiated tumour cells or with one-third of the lymphocytes prepulsed with 100 *μ*M synthetic peptides in optiMEM (Invitrogen, Paisley, UK) for 2 h at 37°C and 5% CO_2_. Restimulated lymphocytes were cultured in RPMI-HEPES medium containing 10% FCS, 2 mM glutamine, combined antibiotics, 1 mM sodium pyruvate, 25 mM HEPES, 5 × 10^−5^ M *β*ME and 1% NEAA for 5 days. Viable cells (effectors) were separated by Lympholyte-M (Cedarlane, Hornby, ON, Canada) gradient, resuspended and admixed at different ratios with 5000 ^35^S-methionine-labelled target cells. Cytolytic assays were carried out as described previously (Carmon *et al*, 2000). Extracts of tumour peptides or normal colon peptides were prepared from a pool of six post-surgical colon carcinoma specimens as described before (Carmon *et al*, 2000).

To determine the threshold for immunogenicity of the individual peptides, we vaccinated two HHD mice with each of the peptides. CTL preparation was made and a cytolytic assay was performed using either the peptide itself or the tyrosinase epitope-loaded target cells as a negative control. We defined the ratio of the specific lysis between the two targets as the ‘immunogenic score’, which expressed as percent of the control lysis. The lysis of the tyrosinase-loaded target cells was between 10–20%. We averaged the control lysis results of all the assays (total 44 measurements in triplicates) and defined the threshold as 95% confidence level above the average. This resulted in an immunogenic score of 125% (a horizontal line at this level was added to Figure 2A). Thus, each peptide that yielded an average immunogenic score of two independent experiments above 125% was rendered immunogenic.

### RNA extraction and real-time PCR

Total RNA from human tumour and normal colon tissues was isolated by TriReagent (Molecular Research Center, Cincinnati, OH, USA) according to the manufacture's instructions. Colon tissues were obtained from Barzilai Medical Center (Ashkelon, Israel) from colon cancer patients according to the Declaration of Helsinki Principles. Reverse transcription was performed on 5 *μ*g of total RNA with an oligo (dT) primer using Superscript™ II (Invitrogen). Relative expression of human *1-8D* gene in human tumour and normal colon tissues was detected using the Roche LightCycler FastStart DNA Master SYBR Green I (Roche, Mannheim, Germany). *β-Actin* gene was used as loading control. Primers were designed to specifically amplify human *1-8D* gene: forward primer for human *1-8D*: 5′-CCTTGACCTGTATTCCACT-3′, reverse primer for human *1-8D*: 5′-GCCATTGTAGAAAAGCGT-3′, these amplify a 102-bp region of the gene. For *β-actin*, forward primer was 5′-GGCATCCACGAAACTAC-3′ and reverse primer 5′-GCTCAGGAGGAGCAAT-3′; these amplify a 209-bp region of the gene. Each reaction was carried out in triplicates in a total volume of 20 *μ*l in glass capillaries containing 5 *μ*l of cDNA sample, 3 mM MgCl_2_, 0.5 mM each primer and 2 *μ*l of LightCycler – FastStart Reaction Mix SYBR Green I mixed with LightCycler FastStart Enzyme. The thermocycling programme consisted of denaturation at 95°C for 10 min, followed by 35–45 cycles of PCR (95°C for 5 s, 60°C for 7 s, 72°C for 11 s and a single fluorescence detection point at 85°C for human *1-8D* and at 87°C for *β-actin*, with transitions programmed at 20°C s^−1^). Melting curves were generated by first heating to 95°C, then cooling to 60°C for 15 s, and finally heating at 0.1°C s^−1^ to 95°C with continuous fluorescence acquisition. Programme was followed by a 30 s 40°C. In each case, product identity was demonstrated by the presence of a single peak on derivate melting curve plots, using the LightCycler software (Roche), and a single band of the appropriate size on gel electrophoresis, which corresponded to the sequence in the database (accession number: X57351).

### Cloning of *1-8D* gene

RNA was isolated from colon tumour specimens using TriReagent (Molecular Research Center). Two micrograms of total RNA was converted to cDNA using SuperScript II (Invitrogen). *1-8D* gene was amplified by PCR by 5′-primer-GGTAAGCTTACCGCCGCTGGTCACCATGAACC; 3′-primer-AGAGCTCGAGGCCTCAATGATGCCTCCTGATCTATCG.

The PCR fragment was cloned into pcDNA3.1/Hygro(+) vector between *Hin*dIII and *Xho*I restriction sites. Myc tag was added at the C terminus by eliminating the stop codon. Positive clones were selected by intracellular staining using Cytofix/Cytoperm Plus kit (Pharmingen, San Diego, CA, USA) using anti-Myc (9E10) mAb (Santa Cruz Biotechnologies, Santa Cruz, CA, USA).

### Adoptive transfer

CD1-nude mice were challenged in the footpad with 3 × 10^6^ HCT/HHD cells. Three days later, 10^7^ viable restimulated effector cells were injected i.v. to the tail vein. One thousand units of IL-2 were injected i.p. twice a day for 7 consecutive days post transfer. Tumour growth was monitored twice a week by measuring the diameter of the footpad with calipers. Mice were killed when one of the tumour diameters reached 10 mm.

### *In vitro* priming of human CTL

Leukapheresis products of three healthy donors were obtained from Barzilai Medical Center according to the Declaration of Helsinki Principles. Peripheral blood mononuclear cells were isolated by centrifugation on Ficoll–Plaque Plus gradients (Amersham, Uppsala, Sweden). The procedure was carried out according to Thurner *et al* (1999) with minor modifications.

## RESULTS

### HCT/HHD and colon carcinoma tumour extracts elicit cross-reactive CTL responses in HHD mice

**Figure 1:**
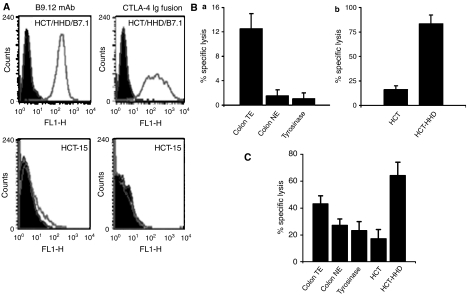
(**A**) HCT/HHD/B7.1 cells highly express HHD and B7.1. Cells were stained with B9.12 or CTLA-4-Ig fusion protein and analysed by flow cytometry. (**B**) HLA-A2.1-restricted and colon-associated lysis induced by the colon carcinoma HCT/HHD transfectant. Mice were immunised with HCT/HHD/B7.1 cells. (a) Lysis of RMA-S/HHD loaded with colon-derived TE, NE and non-relevant peptides (b) as well as the transfectant itself and the parental cell line were monitored by CTL assays. The specific lysis at a 50 : 1 effector to target ratio is shown. (**C**) Colon carcinoma-associated CTL responses in patient-derived TE-immunised mice. CTL assays utilising anti-patient-derived TE-activated lymphocytes were performed. Colon cell line HCT/HHD or non-relevant tyrosinase synthetic peptides as well as tumour- and normal-derived peptide extract loaded on RMA-S/HHD served as targets (NE). The 25 : 1 effector to target ratio is shown.

Successful screening of antigenic peptides relies upon reproducible screening procedures. The CTL responses obtained to vaccinations of the HHD mice with low molecular weight extracts of colon tumours varied from preparation to preparation (data not shown). Therefore, we decided to vaccinate HHD mice with a cellular preparation. After screening several colon carcinoma cell lines, we found that the HCT-15 cell line does not express any HLA class I molecules and therefore may serve as a convenient platform for introduction of the single-chain HHD (Figure 1A, lower left panel).

We established HCT-15 cells that highly express the HHD and murine B7.1 (Figure 1A, upper panels) as potent inducers of CTL responses in HHD mice (Figure 1B, panel b). We tested the ability of CTL raised against HCT/HHD/B7.1 cells to identify antigens extracted from a pool of six colon tumour specimens (tumour extract (TE)). HHD mice were vaccinated with irradiated HCT/HHD/B7.1 cells, spleens were removed and cytolytic activity was assayed on several targets (Figure 1B). We performed a reciprocal experiment, in which HHD mice were vaccinated with RMA-S/HHD/B7.1 loaded with the colon-derived TE and cytolytic activity was measured on the indicated targets (Figure 1C). HCT/HHD/B7.1 elicits a powerful CTL response against itself in an HHD-restricted manner (compare HCT/HHD *vs* HCT). The CTL preferentially identified TE loaded on target cells but not cells loaded with either normal extract (NE) (prepared from a pool of normal colon), or tyrosinase-derived peptide, as a negative control (Figure 1B, panel a). When RMA-S/HHD/B7.1 cells loaded with TE were used to induce a CTL response, HCT was lysed in an HHD-restricted manner. The response mounted against NE and tyrosinase was approximately 20% lower than the response against TE (Figure 1C, *P*<0.05). These experiments demonstrate a significant overlap between HCT antigens and antigens extracted from colon carcinoma tumour specimens. Thus, establishing vaccination with HCT/HHD/B7.1 as an immunological model for human colon carcinoma.

### Twenty-two peptides are antigenic to anti-HCT/HHD/B7.1 effector cells

We focused on putative non-secreted 26 genes (Table 1), previously reported to be overexpressed at least five-fold in colon tumour tissues (Zhang *et al*, 1997). The protein sequences were screened for putative HLA-A2.1-restricted peptides using the ‘independent binding of individual peptide side-chains’ software (Parker *et al*, 1994). We selected high-and medium-affinity HLA-A2.1-restricted peptides (score over 1). Overall, 503 peptides were synthesised by the MPS synthesis technology and loaded on ^35^S-labelled RMA-S/HHD/B7.1 to serve as targets for CTL. Splenocytes of HCT/HHD/B7.1-immunised animals were incubated with the target cells in a 50 : 1 effector to target ratio. Targets that were specifically lysed by more than 10% than the negative control in two consecutive experiments were regarded a positive hit. A list of the antigenic peptides and their position in the protein of their origin is shown in Table 2.

### Seven peptides derived from overexpressed genes are immunogenic in HHD mice

**Figure 2:**
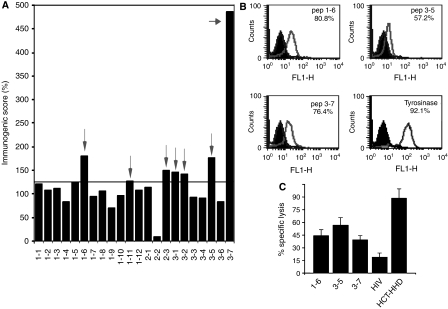
(**A**) Seven peptides are immunogenic. Peptides were loaded on RMA-S/HHD/B7.1, washed and irradiated. For each peptide, two HHD mice were immunised three times as described in Materials and Methods. *In vitro* cytolytic assays were performed on the relevant peptide as target and on an irrelevant peptide, and the immunogenic score was calculated. The positive peptides (above 125%) are marked by arrows. Peptides 1–6, 3–5 and 3–7 are derived from ‘human *1-8D* gene’. (**B**) 1-8D peptides stabilise HLA-A2 on T2 cells. T2 cells were acid stripped and incubated overnight with the indicated peptide and recombinant *β*2 or with *β*2m alone as a negative control. Cells were stained with BB7.2 mAb and analysed by flow cytometry. The number of positive cells above control level is indicated for each of the peptides. (**C**) 1-8D peptides can mount anti-HCT/HHD CTL responses. HHD mice were immunised with HCT/HHD/B7.1 and with peptides loaded on RMA-S/HHD/B7.1. *In vitro* cytolytic assays were performed using HCT/HHD cells as targets. The specific lysis at a 50 : 1 effector to target ratio of a representative experiment out of three is shown.

Next, the antigenic peptides were tested for their specific immunogenicity in HHD mice. HHD mice were immunised with RMA-S/HHD/B7.1 cells loaded with the individual peptides. A cytolytic assay was performed on the peptide itself loaded on labelled RMA-S/HHD or tyrosinase peptide, and the immunogenic score was determined. These experiments were repeated and the average score obtained for each peptide is shown in Figure 2A. Threshold was set as explained in Materials and Methods. According to this criterion, seven peptides showed significant immunogenicity in the HHD mice. Three out of the seven peptides, 1–6, 3–5 and 3–7, which showed the highest immunogenicity, were derived from ‘human *1-8D* gene from interferon inducible gene’ (*1-8D*).

### The peptides of ‘*1-8D gene*’ induce anti-HCT/HHD CTL responses

Intrigued by this finding, we decided to focus on the 1-8D peptides. Using an HLA-A2 stabilisation assay on T2 cells (Firat *et al*, 1999), we verified the binding of the 1-8D peptides to HLA-A2.1 (Figure 2B), the 1-8D peptides bind to HLA-A2.1, although to a lower extent than for the tyrosinase peptide, a strong HLA-A2.1 binder.

We next tested 1-8D peptides for induction of anti-HCT/HHD CTL responses. HHD mice were vaccinated with the 1-8D peptides as previously described (Carmon *et al*, 2000). As a negative control, we used an HIV-derived peptide, TLNAWVKVV. 1-8D peptides mount more than twice higher CTL responses than the HIV peptide at a 50 : 1 effector to target ratio (Figure 2C). We conclude that 1-8D peptide elicits anti-HCT CTL responses.

### The *1-8D* gene is overexpressed in primary colon tumour

**Figure 3:**
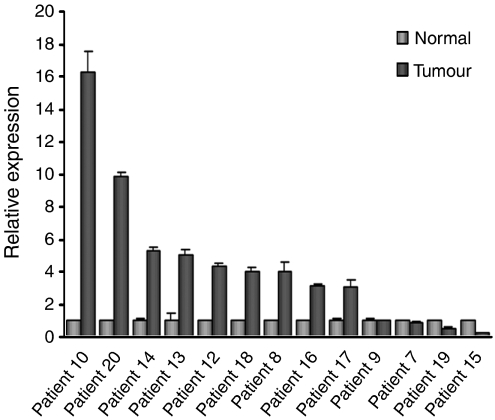
The *1-8D* gene is overexpressed in tumour colon tissues. The relative expression of human *1-8D* gene in tumour and normal colon tissue was determined using real-time PCR. Expression level of *β-actin* was considered equal in both normal and tumour samples and was used for normalisation while calculating the expression level of the *1-8D* gene. Average±s.d. is presented.

The relative expression of the human *1-8D* gene in colon tumour tissues *vs* normal tissues was measured by real-time PCR. An internal standard curve was generated by amplification of four-fold dilution of cDNA for each gene in each run. Based on the cycle number, when the fluorescence detected increased over the noise or base line (crossing point), the linear regression curve was calculated. The correlation coefficient *r* was −1.00, the error was as well minimal, and the slope near to −3.33. Expression level of *β-actin* was used for normalisation. Results are expressed as relative expression level for human *1-8D*. Human *1-8D* gene is overexpressed in 9 samples out of 13 that were tested (Figure 3). These findings indicate that the human *1-8D* gene is a *bona fide* overexpressed gene in tumour colon tissues.

### 1-8D peptides are presented on EL4/HHD/1-8D-Myc

**Figure 4:**
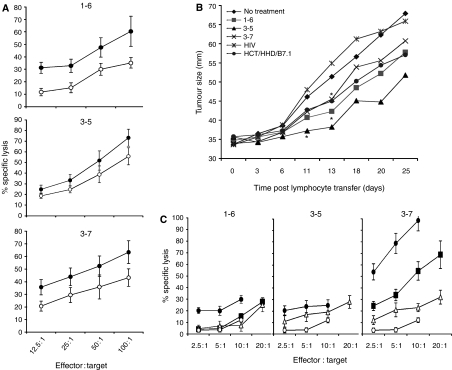
(**A**) 1-8D peptides mount CTL responses that lyse 1-8D transfectants. Each peptide was loaded on RMA-S/HHD/B7.1 and used to immunise HHD mice. *In vitro* cytolytic assays were performed using EL4/HHD cells (open circles) and EL4/HHD/1-8D-transfected cells (dark circles) as a target. The specific lysis of a representative experiment out of three is shown. (**B**) HCT/HHD growth is inhibited by vaccination with 1-8D peptides. Nude mice were challenged in the footpad with HCT/HHD cells. Lymphocytes from vaccinated HHD mice were transferred together with IL-2 to the tumour-bearing mice and the growth of the tumour was monitored twice a week. Statistical significance was achieved for peptides 1–6 and 3–7 from day 13 and for 3–5 treatment from day 11 (Student's *t*-test, *P*<0.05). (**C**) 1-8D peptide primes *in vitro* normal CTLp. Peripheral blood mononuclear cells of three leukapheresis samples were isolated and *in vitro* priming was performed with peptide-pulsed autologus DC. Peripheral blood mononuclear cells were supplemented with IL-7, and 2 days later IL-2 was added and renewed every 3 days. The rest of the stimulations were performed every 7 days over peptide-pulsed monocytes. Seven days after the third stimulation, lymphocytes were harvested and a cytolytic assay was performed using peptide-pulsed or non-pulsed T2 cells as targets. ▪, CTLp from donor A; •, CTLp from donor B; ▵, CTLp from donor C; and ○, non-pulsed T2 cells.

To demonstrate that the 1-8D protein is processed and the peptides are presented, we cloned the *1-8D-Myc* into a pcDNA3.1. vector. Stable EL4/HHD/1-8D cells were generated. HHD mice were immunised with the 1-8D peptides and a cytolytic assay was performed using EL4/HHD/1-8D transfectant and the parental EL4/HHD cells as targets. The *1-8D* transfectant was lysed better than the parental cells when 1–6 and 3–7 restricted CTL were used (Figure 4A, upper and lower panels). CTL restricted to 3–5 show minor selectivity towards the *1-8D* transfectant (Figure 4A, middle panel). We conclude that 1-8D is processed, and peptides 1–6 and 3–7 are presented at the cell surface. These results do not confirm or disprove presentation of 3–5.

### Adoptively transferred anti-1-8D peptide lymphocytes inhibit the growth of HCT/HHD in nude mice

To check whether activation of lymphocytes induced by the 1-8D peptides harbours an immunotherapeutic potential, HCT/HHD cells were inoculated to the footpad of nude mice. Three days post footpad inoculation, restimulated HHD lymphocytes were transferred i.v. Transfer of anti-1-8D peptide CTL caused significant retardation in tumour growth. Retardation was detected for peptide 3–5 starting from day 11 (*P*<0.05), and for peptides 1–6 and 3–7 starting from day 13 (*P*<0.05) applying one-tail Student's *t*-test (Figure 4B).

### Peptide 3–7 activates peripheral CTL precursors in normal human PBMCs

Finally, we checked peripheral blood of three healthy individuals for the presence of CTL precursors (CTLp). Peptide 3–7 primed effectively CTL in lymphocytes from donors A and B and to a lesser extent in lymphocytes from donor C. Peptide 1–6 primed CTL in lymphocytes of donor B alone and peptide 3–5 primed CTL in lymphocytes of donors B and C. Unfortunately due to paucity of PBMCs of donor A, this was not tested with peptide 3–5 (Figure 4C).

## DISCUSSION

One approach to identify human tumour-associated HLA class I-restricted peptides utilises CTL lines derived from patients (Heidecker *et al*, 2000). This approach suffers from several caveats: establishment of CTL lines is a complicated procedure; CTL lines derived from cancer patients may represent the repertoire of an anergised immune system; finally, propagation of CTL lines *in vitro* might reflect sporadic clones surviving culture conditions rather than authentic antitumour clones. In order to bypass these pitfalls and to obtain pure HLA-A2-restricted CTL responses, we used the HHD mice (Pascolo *et al*, 1997). We combined a reverse immunology approach together with a screening procedure that utilises HHD lymphocytes to identify immunodominant epitopes of colon carcinoma-overexpressed genes.

The HCT-15 cell line that possess high-tumorigenic properties was modified to be a suitable antigen-presenting cell by transfection with the single-chain HHD and the costimulatory B7.1 molecule (Cayeux *et al*, 1997) (Figure 1A). These cells were used to immunise HHD mice, as a source of CTL for epitope screening. It was important to demonstrate that HCT/HHD/B7.1 elicits a CTL response that overlaps the response obtained with TE to ensure physiological significance of the screen (Figure 1B).

We found that HCT/HHD/B7.1 elicits strong anti-self, HHD-restricted CTL response that recognises preferentially the TE (Figure 1B). Furthermore, CTL of mice, immunised with RMA-S/HHD/B7.1 cell-loaded TE, show high-specific lysis (over 60%) of HCT/HHD. The lysis was five-fold higher than of parental cells. These results suggest that processing and presentation of HLA-A2.1-restricted and colon-associated peptides are performed by the HCT/HHD colon carcinoma cells. Likewise, the high-specific lysis of HCT/HHD transfectants indicates an overlapping peptide repertoire between the TEs and the cell line.

Two-fold preferential lysis of colon-derived tumour peptide- *vs* normal tissue peptide-loaded targets was observed upon vaccination with colon TE peptides. This observation supports the existence of a window of specificity, towards which tumour immunotherapy can be directed.

We selected 26 genes of putative non-secreted proteins that are expressed at least five-fold higher in tumours than in normal colon (Table 1). These genes were analysed for HLA-A2.1-restricted peptides. We screened over 500 HLA-A2.1-restricted peptides and we found that in a cytolytic assay lymphocytes recognised 22 peptides, rendering them antigenic to the lymphocytes.

Next, we narrowed down the antigenic peptides to those that are also immunogenic. HHD mice were vaccinated with each of the individual peptides, and lymphocytes were assayed for lysing target cells, loaded with either the peptide itself or an irrelevant peptide. Interestingly, only seven peptides could mount a significant CTL response in HHD mice (Figure 2A). Three of the seven were derived from the 1-8 family of interferon inducible proteins, highlighting these gene products as novel TAA. Binding of the peptides to HLA-A2 was confirmed by performing a stabilisation assay on T2 cells (Figure 2B).

The *1-8* gene family is inducible by both type I and type II interferons (Lewin *et al*, 1991). Three members of the family: *1-8D*, *1-8U* and *9-27* (also called *IFITM2*, *IFITM3* and *IFITM1*, respectively) are linked on an 18-kb fragment of chromosome 11 and are highly homologous (Lewin *et al*, 1991). The *9-27* was shown to be an integral membrane protein identical to the Leu-13 antigen. It forms a complex with other proteins and is implicated in cell adhesion and growth inhibitory signals (Deblandre *et al*, 1995). The *1-8U* is expressed in colitis-associated colon cancer and in severely inflamed mucosa in ulcerative colitis (Hisamatsu *et al*, 1999), as well as in an androgen-independent variant of the prostate carcinoma line LNCaP, but not in an androgen-dependent variant (Vaarala *et al*, 2000). Interestingly, *1-8D* and *9-27* are induced by radiation in a p53-negative line, in an interferon-independent mechanism (Clave *et al*, 1997). The expression of *1-8* gene family is induced in a human adrenocortical carcinoma cell line in response to angiotensin II treatment, implicating other signalling pathways to control their expression (Daido *et al*, 2001). Moreover, it has been demonstrated that *IFITM1* activity is required for primordial germ cell (PGC) transient from the mesoderm into the endoderm and that *IFITM3* is sufficient to confer autonomous PGC-like homing properties to somatic cells (Tanaka *et al*, 2005).

Mouse *1-8* genes termed *fragilis/mil* have been implicated in the acquisition of germ cell competence and specification (Barlow *et al*, 1984; Saitou *et al*, 2002; Lange *et al*, 2003). Sequence similarity of human, murine and rat *1-8D* genes is high (68%). The homology between human 1-8D and the murine 1-8D proteins (*m1-8D*, accession number: BC010291) is 78%. The epitopes identified from the human 1-8D protein (1–6, 3–5 and 3–7 peptides) are highly similar to the corresponding peptides in m1-8D protein: human and murine 1–6 (EMLKEEQEV *vs* ERIKEEYEV), human and murine 3–5 (LILGIFMTI *vs* LVLSILMVV), and human and murine 3–7 (KCLNIWALI *vs* KCLNISTLV), respectively.

We further characterised the 1-8D peptides as potential antigens for vaccination against colon cancer. Curiously, peptide 3–7, the most immunogenic peptide in HHD mice harbours a cysteine residue in its sequence. Moreover, the cysteine residue is situated at the anchor position of HLA-A2.1 restriction pattern, which shows preference to aliphatic amino acids like leucine. In congruent to the study of Chen *et al* (1999), mutation of the cysteine to leucine enhanced the immunogenicity of the peptide albeit not affecting the binding to HLA-A2.1 (data not shown).

An anti-HCT/HHD CTL response was obtained upon vaccination of HHD mice with either of 1-8D peptides with respect to the HIV-derived peptide (Figure 2C). The recognition of HCT/HHD by anti-1-8D lymphocytes further supports the hypothesis of 1-8D immunodominance in this model.

We elaborated our analysis to tumour specimens and found *1-8D* to be overexpressed in 9 out of 13 fresh tumour samples as compared to adjacent normal tissue using real-time PCR (Figure 3). These results establish *1-8D* as a *bone fide* overexpressed gene in colon cancer.

EL4/HHD cells were transfected with *1-8D*. HHD mice were vaccinated with either of the 1-8D-derived peptides and the susceptibility of EL4/HHD and EL4/HHD/1-8D cells for lysis by the active lymphocytes was measured. Despite the high background, the *1-8D* transfectant was better lysed than EL4/HHD cells, by CTL restricted to peptides 1–6 and 3–7, while peptide 3–5 did not show 1-8D preference. This fact indicates successful processing and presentation of 1–6 and 3–7. Presentation of 3–5 was not confirmed by this assay (Figure 4A). Mixing the anti-1-8D peptide lymphocytes prior to incubation with the targets did not improved the lysis of the target cells, but rather resulted in an average lysis (data not shown). This observation suggests that the three peptides are derived from a single protein, and presented on MHC class I at similar levels.

We further examined whether activation of lymphocytes induced by the 1-8D peptides harbours an immunotherapeutic potential. We established an adoptive transfer model in which CTL to 1-8D peptides retarded the growth of HCT/HHD (Figure 4B). In this experiment, peptide 3–7 was found to be the least effective, although statistical significance between the various 1-8D peptides was not achieved. Nonetheless, this result is in concordance with the *in vitro* cytolytic capacity of HCT/HHD that ranked peptide 3–5 as the most effective vaccine against HCT/HHD (Figure 2C).

Owing to the strong genetic and familial aetiology of colon cancer, this cancer is appropriate to prophylactic treatment (Severin, 1999; Lindblom, 2001). Hence, we were interested to assess the potential of 1-8D peptides to prime peripheral CTL of normal HLA-A2.1-positive donors. Peripheral blood mononuclear cells of three normal donors were isolated and assayed for the existence of CTLp by *in vitro* stimulations with autologus DCs. Peptide 3–7 was found to be highly efficient in activating peripheral CTLp to lyse target cells in a peptide-specific manner for donors A and B and to a lesser extent for donor C. Peptide 1–6, however, primed CTL in donor B alone and peptide 3–5 primed CTL in donors B and C, yet with less efficiency than peptide 3–7 (Figure 4C). This might reflect specific gaps in the T-cell repertoire in the donors' peripheral blood that might result from expression of additional HLA alleles. CTL precursors for all peptides were found in the peripheral blood of donor B, indicating no tolerance to peptides 1–6 and 3–5 (Figure 4C). Peptide 3–7 is shared between the three members of the *1-8* gene family, while the other peptides (1–6 and 3–5) are unique for the *1-8D* gene. The high immunogenicity of peptide 3–7 in the peripheral blood might be influenced from the contribution from the three distinct genes, as opposed to the two other peptides that derive from a single gene. Additional experiments with PBMCs of different donors are needed to account for immunogenicity of the 1-8D peptides in human.

Our study unravels for the first time immunodominant CTL epitopes of the *1-8D* gene family. Based on the interferon regulation of these genes, and the tight connection of colon carcinogenesis to inflammation (Lewin *et al*, 1991), we propose the *1-8D* genes as potential targets for a CTL-mediated immunotherapy of colon carcinoma.

## Figures and Tables

**Table 1:** Colorectal-associated genes

**Gene no.**	**Gene name**	**No. of HLA-A2.1 peptides**
1	Human defensin 6	11
2	Human ADP/ATP translocase	24
3	Human parathymosin	1
4	Human 1-8U gene from interferon inducible gene	25
5	Human chaperonin-like protein	29
6	Human SPARC/osteonectin	23
7	Human 1-8D gene from interferon inducible gene	24
8	Human TB2 gene	29
9	Human alpha-1 collagen	12
10	Human mRNA for dipeptidase	19
11	Fibronectin	38
12	Actin binding protein	39
13	HCG IV mRNA	19
14	HLA-DR antigens associated invariant gamma chain	19
15	MHC class I HLA-C.1 gene	29
16	polyA binding protein	29
17	Transforming growth factor-*β* induced gene	19
18	*H. sapiens* mRNA for laminin-binding protein	18
19	Human mRNA sequence	14
20	Insulin like growth factor II	19
21	Human ribosomal protein L23a mRNA	5
22	Human acidic ribosomal phosphoprotein P1	8
23	Human liver mRNA fragment DNA binding protein UPI	5
24	Ribosomal protein L37	1
25	Human MHC protein homologous to chicken B complex	29
26	HB23 gene for B23 nucleophosmin	15

The genes were selected according to the following rules: (1) candidate genes with secretion sequences were excluded; (2) candidate gene must be overexpressed in tumours at least five-fold higher than in normal tissue.

HLA-A2.1-restricted peptides from the selected genes were selected according to their consensus binding motifs.

**Table 2:** List of antigenic HLA-A2.1-restricted peptides, their respective gene and position

**Peptide serial no.**	**Name of gene**	**Position**
1-1 VLYDELKKV	Human ADP/ATP translocase	253–261
1-2 LLVIIPVLV	Human 1-8D gene from interferon inducible gene	119–127
1-3 VQPQSPVAV	Actin binding protein	10–18
1-4 FELAAESDV	Transforming growth factor β induced gene	380–388
1-5 GQQSTVSDV	Actin binding protein	1415–1423
**1-6 EMLKEEQEV**	Human 1-8D gene from interferon inducible gene	20–28
1-7 IQQYGHQEV	Actin binding protein	657–665
1-8 ALRGHSHFV	Human MHC protein homologous to chicken B complex	58–66
1-9 VIATNILLV	Human chaperonin-like protein	368–346
1-10 TILTAVLLV	Human defensin 6	5–13
1-11 IVDDITYNV	Actin binding protein	492–500
1-12 TLQLSRAPV	Human mRNA for dipeptidase	223–231
2-1 ALPDETEVV	Human osteonectin	23–31
2-2 IPMGKSMLV	Insulin like growth factor II	3–11
2-3 KIEDNNTLV	Human ribosomal protein L23a mRNA	89–97
3-1 MLTINGKAI	Transforming growth factor *β* induced gene	343–351
3-2 SIAEFFSDI	Human TB2 gene	105–113
3-3 ALGFYPAEI	Human thyroid hormone binding protein (p55)	229–237
3-4 WVVYGVFSI	Human TB2 gene	98–106
**3-5 LILGIFMTI**	Human 1-8D gene from interferon inducible gene	110–118
3-6 DLQETLVKI	Human chaperonin like protein	316–324
**3-7 KCLNIWALI**	Human 1-8D and 1-8U genes from interferon inducible genes	103–111

The 1-8D-derived peptides are bold and underlined.
